# An integrated dataset of near-surface Eulerian fields and Lagrangian trajectories from an ocean model

**DOI:** 10.1038/s41597-024-03813-z

**Published:** 2024-08-29

**Authors:** Shane Elipot, Eli Faigle, Brian K. Arbic, Jay F. Shriver

**Affiliations:** 1 Rosenstiel School of Marine, Atmospheric, and Earth Science, University of Miami, Miami, FL 33149 USA; 2 Department of Earth and Environmental Sciences, University of Michigan, Ann Arbor, MI 48109 USA; 3 Ocean Sciences Division, Naval Research Laboratory, Stennis Space Center, MS 39529 Mississippi, USA

**Keywords:** Physical oceanography, Scientific data

## Abstract

A dataset consisting of numerically simulated oceanic velocities and sea surface height changes, provided conjointly from Eulerian and Lagrangian points of view, is made available as cloud-optimized archives on a cloud storage platform for unrestricted access. The Eulerian component of the dataset comprises oceanic velocity components at 0 m and 15 m depth, as well as total and steric sea surface height changes, obtained at hourly time steps for one year, with an approximate horizontal resolution of 1/25 degree on an irregular global geographical spatial grid, from the HYbrid Coordinate Ocean Model. The Lagrangian component of the dataset comprises the trajectories of particles advected in the Eulerian velocity field of the model. The particles were advected forward and backward for 30 days from a regular 1/4 degree grid in order to achieve 60-day long trajectories at 0 m and 15 m depths, with start times separated by 30 days, in 11 releases. This integrated dataset may help to link Eulerian and Lagrangian observational perspectives.

## Background & Summary

The dataset presented^1^ consists of numerically simulated oceanic velocities and sea surface height changes, provided conjointly from Eulerian and Lagrangian points of view. The overarching goal for these data is to be used by oceanographers and climate scientists who want to seek answers to questions related to near-surface ocean motions and sea level changes. Examples of such questions include: What are the spatial and temporal scales of oceanic currents variability? How does sea level vary in different oceanic regions? How do pollutants, plastics, organic or non-organic particles get dispersed and transported in the ocean? Another category of questions, which may be answered by this dataset because it provides both Eulerian and Lagrangian perspectives, is related to the way the global ocean is currently observed, or how it can be better observed in the future. For example, can we use freely drifting buoys to accurately estimate the kinetic energy of ocean currents? Can surface drifting buoys be equipped with global navigation satellite system to measure sea level changes^2^?

Because the global ocean cannot be observed everywhere all at once, oceanographic research notably relies on the data from numerical simulations to further understand the real ocean. The specific code, or ocean model, that generated the data comprising this current dataset, the HYbrid Coordinate Ocean Model (HYCOM), is publicly available (www.hycom.org). However, actually running the model and saving its Eulerian outputs, as well as generating the subsequent Lagrangian dataset described here, requires high performance computing and large physical storage, which are not commonly available. Therefore, this dataset aims at democratizing this advanced tool by being described here and by being available on a freely accessible cloud storage platform. A similar Eulerian dataset, emanating from another state-of-the-art ocean model (MITgcm, see^3^), is available through a data portal of the National Aeronautics and Space Administration (data.nas.nasa.gov/ecco/eccodata/llc_4320/). A comparison of these HYCOM and MITgcm simulations has been recently conducted to evaluate their respective strengths and limitations^4^.

This dataset presents an innovative integration of both Eulerian and Lagrangian perspectives on oceanographic data, thereby offering an enhanced framework for oceanographers to analyze and interpret ocean dynamics. The Eulerian view, derived from a conventional gridded representation native to HYCOM, and the Lagrangian view, based on simulated particle trajectories, are juxtaposed to provide comprehensive insights into oceanographic phenomena from fixed and mobile observation platforms. Such platforms include satellite observations and moored instruments, as well as drifting devices like surface buoys.

The dataset’s novelty extends beyond its availability to its true accessibility. The two components of the dataset, the Eulerian and the Lagrangian, are hosted on a single cloud platform (Amazon S3), and are formatted with *zarr* (zarr.readthedocs.io) to optimize cloud-based access and analyses. The Eulerian component of this dataset has already been available, yet from a University server requiring to log in and as 17,519 individual NetCDF files, and thus has not been amenable to facile access and analysis. In contrast, the Lagrangian component of the dataset represents a completely novel dataset component and has not been made available previously. This integrated dataset may help to link Eulerian and Lagrangian observational perspectives, and to enhance the accessibility and utility of oceanographic data for scientific research.

## Methods

### HYCOM simulation

The ocean velocity components (zonal and meridional) at 0 m and 15 m depth, and sea surface height (SSH) variables (total and steric), are from the outputs of a 1-year simulation (calendar year 2014) of the HYbrid Coordinate Ocean Model (HYCOM), run in a non-assimilative configuration. Previous publications describe the underlying dynamical core^5^ and the details of the simulation used here^4^ and are succinctly reported here. This HYCOM simulation is global, with an approximate horizontal resolution of 1/25 degree (4.4 km at the Equator), includes 41 hybrid vertical layers, and is integrated with a 75-second baroclinic time step^5^. HYCOM utilizes terrain-following coordinates in coastal regions and isopycnal coordinates in open ocean regions. In the upper open ocean, HYCOM comprises 14 z-coordinates layers with thickness varying between 1.00 m and 6.87 m within the top 30 m of the ocean. Ocean velocity at the surface, or 0 m, actually refers to velocity of the top layer of the model which has a thickness of 1 m. The 15 m ocean velocities were derived by interpolating linearly the velocities of the two layers centered at 13.185 m and 18.55 m depths. Such depth levels were originally chosen with the purpose of comparing the modeled velocities to the data of the Global Surface Drifter Array/NOAA Global Drifter Program^4,6^. This program maintains an array of approximately 1300 surface drifters equipped with a drogue centered at 15 m depth and their displacements are expected to be representative of oceanic velocities at that depth. Once the drogue of a drifter detaches, its displacements are closer to be representative of oceanic velocities at the surface^7^.

HYCOM uses the *K* profile parameterization (known as KPP) for vertical mixing within the ocean surface boundary layer^8^. This HYCOM simulation is forced by 3-hour atmospheric fields from the U.S. Navy Global Environmental Model^9^, converted to surface fluxes using bulk formulas^10^. The wind stress is calculated using 10-m wind speed relative to the model surface ocean speed. This HYCOM simulation includes tidal forcing with the two largest diurnal components (K_1_ and O_1_) and the three largest semidiurnal components (M_2_, S_2_, and N_2_). The Self-attraction and Loading forcing term^11^ is applied from the altimetry-constrained TPXO8 barotropic tide model^12^. The HYCOM parameterized topographic wave drag scheme^13^ is tuned to minimize the M_2_ surface elevation errors with respect to TPXO8.

**Fig. 1:**
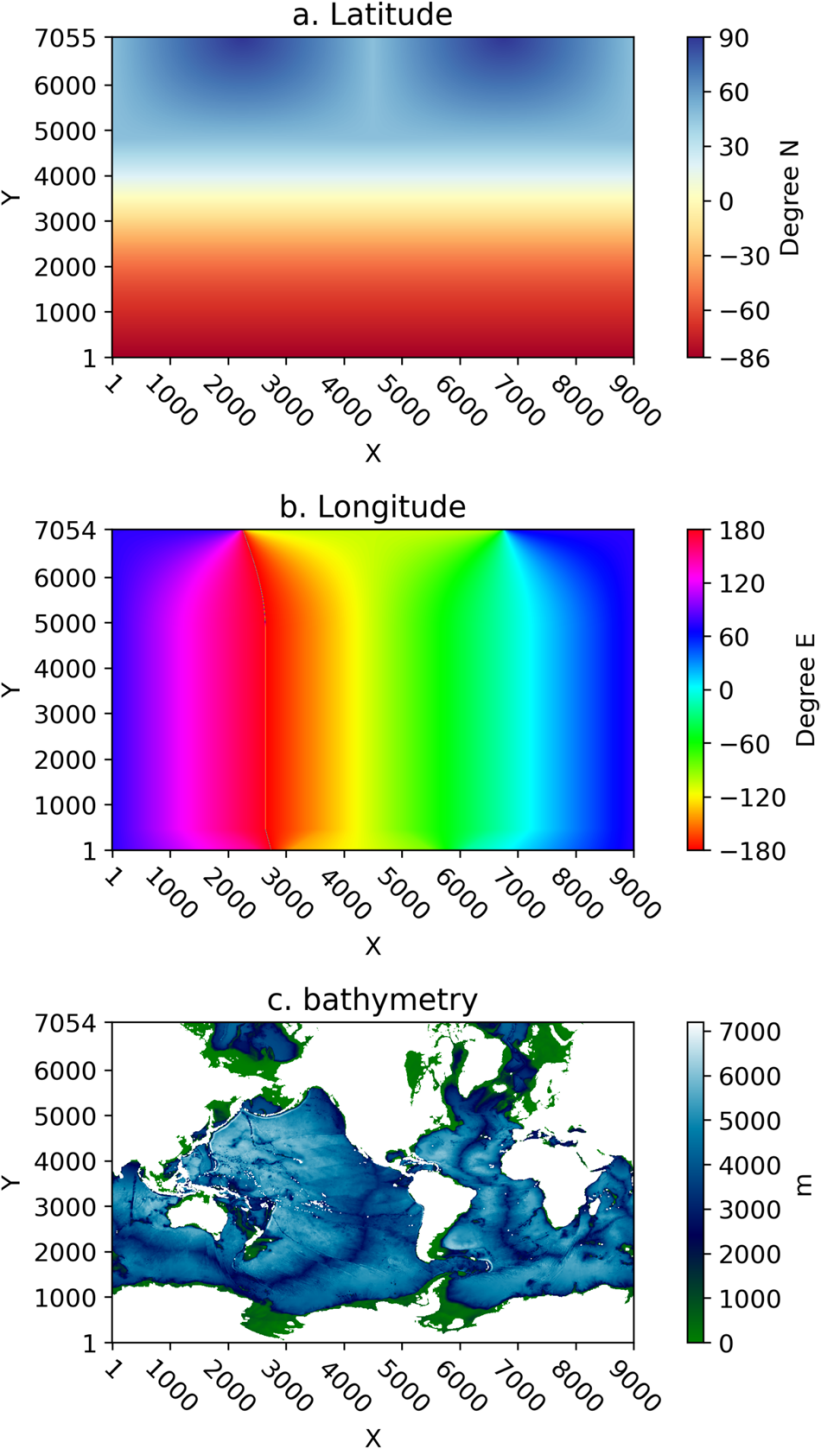
HYCOM Latitude (**a**), Longitude (**b**), and Depth (**c**) as a function of the (*X*, *Y*) grid coordinates with dimensions 9000 and 7055.

HYCOM uses a discretization of the globe with a C-grid for which ocean velocity components are defined on staggered grids, and SSH variables are defined on a grid interspaced between the velocity grids. For this dataset, however, outputs of the model were interpolated and archived on a common (*X*, *Y*) spatial grid with dimensions (9000, 7055). The latitude and longitude of the points on this grid are non-regular, placing two “north poles” on the American and Eurasian continental masses. As such, the latitude is not constant along the *X* direction at high *Y* values, and the longitude is not constant along the Y direction at high and low *Y* values. Figure 1 displays the latitude, longitude, and model depth values on this geographically non-regular grid.

### Particle trajectory simulations

#### Release strategy and trajectory assembly

Using the *Ocean Parcels* Python software version 2.4.1.dev9^14,15^, we released numerical particles in the 0 m and 15 m hourly velocity fields of the HYCOM simulation. We devised a release strategy in order to minimize sampling biases that may occur in horizontally divergent regions of the ocean. Using hourly velocity fields, the particles were advected with an integration time step of 20 minutes forward in time for 30 days, as well as backward in time for 30 days, from common release points. These points define a regular geographical grid at 1/4 degree intervals, from −180 degrees E to 179.75 degrees E in longitude, and from −70 degrees N to 75 degrees N in latitude. Forward and backward advections were conducted at both 0 m and 15 m depths, starting from 2014-01-31 01:00:00 and subsequently every 30 days until 2014-11-27 01:00:00, thus forming 11 releases. Particle positions were output and saved at the top of each hour. Next, we assembled the forward and backward trajectories to form 60-day long (or 1440-hour long) physically-consistent particle trajectories. As a consequence, after 30 days from the common date and time start of each release experiment, the assembled trajectories are such that all particles are simultaneously located on the regular 1/4 degree grid. As the particles were being advected by *Ocean Parcels*, they sampled the model depth and the time-varying model SSH and steric SSH. Particles velocities were subsequently calculated at every time step with a forward difference scheme using the *clouddrift* Python software^16^.

The number of particles successfully and originally released at 0 m and at 15 m for each of the 11 releases differs slightly because of the hypsometric changes of the model between these two depth levels, and because particles were retained upon release depending on their initial speed (Eulerian speed interpolated at release locations less than 1 × 10^−12^ m s^−1^ was used to detect and discard particles effectively released on land). More particles were successfully released at 0 m compared to 15 m because the ocean surface area is larger at 0 m compared to 15 m. We formed a consistent dataset within each release depth by keeping only the particles for which we found common identification numbers (*id* variable, as assigned by *Ocean Parcels*) across all 11 temporal sets. We identified 593,292 and 587,225 trajectories with common *id* numbers at 0 m and 15 m, respectively. All particle *id*s in the 15-m sets can also be found in the 0-m sets because numerical *id*s are assigned by *Parcels* based on the common release arrangement for the forward and backward experiments. As an example, because the effective release location of the particles is the middle step of the assembled trajectories (720 out of 1440), all particles with *id* 105763 are located at longitude −134.5 and latitude −64.75 at time step 720 (719 for 0-based indices) of their respective 1440-step trajectories.

#### Mask definition for grounded particles

We include with the particle trajectory data a determination of particle grounding, also known as beaching, when a particle is advected with infinitesimally decreasing speed towards a coastal boundary of the HYCOM model where the velocity is null. This occurred in our experiments because we did not implement any specific boundary condition along coastal grid cells to prevent grounding. Because the particle advections were conducted forward in time for 30 days and backward in time for 30 days from a regular grid on the sphere, particles could become grounded either at the beginning or at the end of the 60-day long assembled trajectories. We proceed to determine grounding as follows, based on a trial and error approach. For grounding occurring during the 30-day forward advection period, we look for the first period longer than 24 hours during which a particle speed is less than 0.003 m s^−1^, equivalent to a displacement less than 259.2 m over 24 hours. We then take the first time step of that 24-hour period to be the grounding time. This time and all subsequent times are therefore flagged as grounded. For grounding occurring during the 30-day backward advection period, we proceed with the same algorithm but reversed in time: we look for the last period longer than 24 hours during which a particle speed is less than 0.003 m s^−1^. We then take the last time step of that 24-hour period to be the grounding time. This time and all previous times are therefore flagged as grounded. Relatively few particle locations in the dataset are flagged as grounded following the scheme just described. Grounding occurs in about 5% of the trajectories for 1.4% of the total number of data points at 0 m, and in about 2.5% of the trajectories for 0.7% of the total number of data points at 15 m.

## Data Records

The dataset version 1.0 has been deposited on Zenodo at 10.5281/zenodo.12796468^1^. The data files are hosted on the Registry of Open Data on Amazon Web Services (AWS), for which the RODA page is https://registry.opendata.aws/hycom-global-drifters/. The files are stored in an AWS S3 bucket with Amazon Resource Name (ARN) arn:aws:s3:::hycom-global-drifters. A “README” file, directly accessible at https://hycom-global-drifters.s3.amazonaws.com/README.txt, provides descriptions of the files within the S3 bucket.

### Eulerian files

The Eulerian fields of velocity components and total and steric SSH variables are originally available as NetCDF files through the OSiRIS/Globus infrastructure^17^. From this archive, 8759 individual files for velocity and 8759 individual files for SSH can be downloaded, totaling approximately 28TB. Each file corresponds to one hourly time step, from 2014-01-01 01:00:00 to 2014-12-31 23:00:00, with each variable provided on the non-regular (*X*, *Y*) grid. Because this archival structure is not amenable to facile analyses along the time dimension, we reformatted these data utilizing the capabilities of the *zarr* file format (zarr.readthedocs.io) which has been designed for the storage of chunked and compressed multidimensional data. This format is well-suited for cloud computing and storage systems such as the AWS S3 bucket used for this dataset.

Python scripts utilizing the *xarray* package (docs.xarray.dev) were used to divide and combine the individual NetCDF files from OSiRIS^17^ into 12 zarr stores for the velocity variables and 12 zarr stores for the SSH variables. Specifically, we combined the 8759 hourly time steps of the data into 11 consecutive sets of 720 hour or 30 days, and one final and twelfth set of 839 hours (34 days and one hour) to complete the time period. Zarr stores can hold large data arrays by breaking them up in smaller arrays, or chunks, which are contiguously stored in computer memory, and which can be handled independently when performing parallel computations. As such, the zarr stores were further processed using the Python *rechunker* package (rechunker.readthedocs.io). From the original chunking of the NetCDF files, we achieved in general (720, 1, 1, 9000) chunks for variables with dimensions (*time*, *Depth*, *Y*, *X*) and (720, 1, 9000) chunks for variables with dimensions (*time*, *Y*, *X*). As such, the individual zarr stores are optimized for analysis along the *time* and *X* dimensions.

Table 1 lists the characteristics and data variables of the Eulerian zarr stores with names hycom12-x-rechunked-corr.zarr and hycom12-ssh-x-rechunked-corr.zarr, with x from 1 to 12. For these, Table 2 lists the first and last date and time, the number of steps, and the 1-based indices.

**Table 1:** Characteristics and data variables of the Eulerian zarr stores.

Variable	Dimension(s)	Comment
Velocity stores: hycom12-x-rechunked-corr.zarr		
*Depth*	*Depth*	0.0 m or 15.0 m, coordinate with dimension of the same name
*X*	*X*	1 to 9000, no unit, coordinate with dimension of the same name
*Y*	*Y*	1 to 7055, no unit, coordinate with dimension of the same name
*time*	*time*	0 to 719 (or 838), hours since first step, coordinate with dimension of the same name
*Latitude*	*(X,Y)*	degree north
*Longitude*	*(X,Y)*	degree east
*u*	*(time, Depth, Y, X)*	eastward_sea_water_velocity, in meter per second
*v*	*(time, Depth, Y, X)*	northward_sea_water_velocity, in meter per second
SSH stores: hycom12-ssh-x-rechunked-corr.zarr		
*X*	*X*	1 to 9000, no unit, coordinate with dimension of the same name
*Y*	*Y*	1 to 7055, no unit, coordinate with dimension of the same name
*time*	*time*	0 to 719 (or 838), hours since first step, coordinate with dimension of the same name
*Latitude*	*(X,Y)*	degree north
*Longitude*	*(X,Y)*	degree east
*ssh*	*(time, Y, X)*	sea surface height, in meter
*steric_ssh*	*(time, Y, X)*	steric change in sea surface height, in meter
Bathymetry store: hycom12-bathy.zarr		
*X*	*X*	1 to 9000, no unit
*Y*	*Y*	1 to 7055, no unit
*bathymetry*	*(X,Y)*	ocean depth, in meter, positive

The possible dimensions and lengths for the variables are *Depth* (2), *Y* (7055), *X* (9000), and *time* (720 or 839).

**Table 2:** Date and time characteristics of the Eulerian zarr stores.

Store	First Datetime	Last Datetime	Step number	1-based indices
1	2014-01-01 01:00:00	2014-01-31 00:00:00	720	1 to 720
2	2014-01-31 01:00:00	2014-03-02 00:00:00	720	721 to 1440
3	2014-03-02 01:00:00	2014-04-01 00:00:00	720	1441 to 2160
4	2014-04-01 01:00:00	2014-05-01 00:00:00	720	2161 to 2880
5	2014-05-01 01:00:00	2014-05-31 00:00:00	720	2881 to 3600
6	2014-05-31 01:00:00	2014-06-30 00:00:00	720	3601 to 4320
7	2014-06-30 01:00:00	2014-07-30 00:00:00	720	4321 to 5040
8	2014-07-30 01:00:00	2014-08-29 00:00:00	720	5041 to 5760
9	2014-08-29 01:00:00	2014-09-28 00:00:00	720	5761 to 6480
10	2014-09-28 01:00:00	2014-10-28 00:00:00	720	6481 to 7200
11	2014-10-28 01:00:00	2014-11-27 00:00:00	720	7201 to 7920
12	2014-11-27 01:00:00	2014-12-31 23:00:00	839	7921 to 8759

The combined size of the velocity and SSH zarr stores is approximately 5.6 TB, which corresponds to a 65% size reduction compared to the OSiRIS archive. Note that the NetCDF files for velocity on OSiRIS (2024) contain two additional variables, the zonal and meridional components of ocean bottom velocity, which are not included in the zarr archives in the AWS S3 bucket.

A single zarr store hycom12-bathy.zarr is provided for the model on the (*X*, *Y*) grid. Grid points located on land are indicated by Not-a-Number (NaN) values (Fig. 1c).

### Lagrangian files

The Lagrangian dataset is constituted of 22 zarr stores. There are 11 stores for numerical particles advected at the surface of the model (0 m) in stores global_hycom_0m_step_x.zarr with x from 1 to 11, and 11 stores for numerical particles advected at 15 m depth in stores global_hycom_15m_step_x.zarr with x from 1 to 11. Each zarr store contains either 593,292 (0 m) or 587,225 (15 m) particle trajectory data at 1440 hourly time steps forming 60 days. The characteristics and data variables of the Lagrangian zarr stores are listed in Table 3. The start, median, and end dates and times of the 11 sets of trajectories at each depth are listed in Table 4.

**Table 3:** Characteristics and data variables of the Lagrangian zarr stores.

Variable	Dimension(s)	Comment
Stores: global_hycom_[0,15]m_step_x.zarr		
*id*	*traj*	Identification number assigned to a particle by the *Ocean Parcels* software
*obs*	*obs*	0-based index from 0 to 1439, coordinate with dimension of the same name
*depth*	*(traj, obs)*	Model depth interpolated along trajectory
*grounding*	*(traj, obs)*	Boolean variable which is True if the particle is estimated grounded
*lat*	*(traj, obs)*	Latitude, degree north
*lon*	*(traj, obs)*	Longitude, degree east
*ssh*	*(traj, obs)*	Sea Surface Height, in meter
*steric_ssh*	*(traj, obs)*	Steric change in Sea Surface Height, in meter
*time*	*(traj, obs)*	Hours since first step of each set
*ve*	*(traj, obs)*	Eastward particle velocity, in meter per second
*vn*	*(traj, obs)*	Northward particle velocity, in meter per second

The possible dimensions for the variables are *traj* (593292 or 587225) and *obs* (1440).

**Table 4:** Start, middle, and end dates for the 11 Lagrangian sets of particle data at each depth.

Particle set number	Start date	Median date	End date
1	2014-01-01 01:00:00	2014-01-31 01:00:00	2014-03-02 00:00:00
2	2014-01-31 01:00:00	2014-03-02 01:00:00	2014-04-01 00:00:00
3	2014-03-02 01:00:00	2014-04-01 01:00:00	2014-05-01 00:00:00
4	2014-04-01 01:00:00	2014-05-01 01:00:00	2014-05-31 00:00:00
5	2014-05-01 01:00:00	2014-05-31 01:00:00	2014-06-30 00:00:00
6	2014-05-31 01:00:00	2014-06-30 01:00:00	2014-07-30 00:00:00
7	2014-06-30 01:00:00	2014-07-30 01:00:00	2014-08-29 00:00:00
8	2014-07-30 01:00:00	2014-08-29 01:00:00	2014-09-28 00:00:00
9	2014-08-29 01:00:00	2014-09-28 01:00:00	2014-10-28 00:00:00
10	2014-09-28 01:00:00	2014-10-28 01:00:00	2014-11-27 00:00:00
11	2014-10-28 01:00:00	2014-11-27 01:00:00	2014-12-27 00:00:00

The zarr chunks for the data variables are (23301, 1440) except for *grounding* which are (93206, 1440) and *time* which are (9176, 45). As such, the data are generally optimized for analysis along the *obs* dimension, that is along trajectories (or equivalently time).

The coordinate variable *id* is a tagging number assigned to a particle by the *Ocean Parcels* software. The same id numbers are all found in each of the 11 sets at 0 m, and the same is true for the 11 sets at 15 m. There are 587,225 common ids between a set at 0 m and a set at 15 m.

## Technical Validation

This HYCOM Eulerian dataset of 0 m and 15 m velocities has been previously compared to the same depth velocities of another global numerical model of comparable characteristics (the Massachusetts Institute of Technology general circulation model or MITgcm), as well as against in situ estimates of oceanic velocities from the drogued and undrogued drifters of the NOAA Global Drifter Program^4^. This three-way comparisons, two numerical global models and one in situ global dataset, cannot provide a strict and complete validation of the HYCOM velocity dataset for reasons too numerous to list here. Yet, the comparison of^4^ demonstrated the reliability and usefulness of the HYCOM Eulerian velocity dataset to investigate a variety of oceanic processes.

**Fig. 2:**
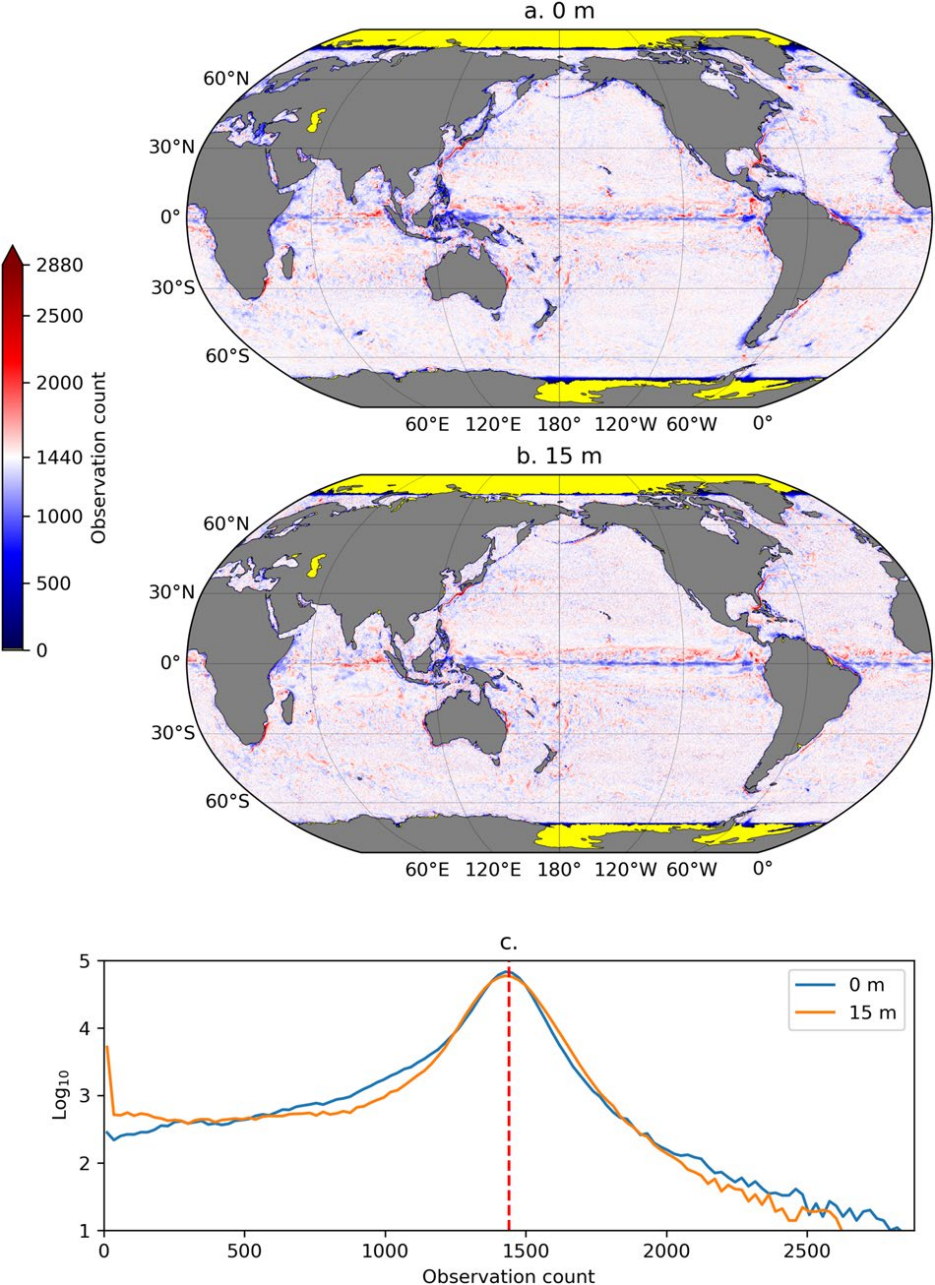
Count of hourly Lagrangian particle data at 0 m (**a**) and at 15 m (**b**) in 1/4 degree longitude-latitude bins, averaged over the 11 Lagrangian time periods of the dataset. Panel c shows the histograms of count values between 70°S and 75°N at both 0 m and 15 m (on a decimal logarithmic scale). The red vertical dashed line in this panel indicates 1440, the expected count in 1/4 degree bins if the particles were immobile.

We now turn to validating the Lagrangian dataset. Figure 2 displays the count of Lagrangian particle locations, or particle density, per 1/4 degree bins, on average, for the 11 sets of trajectory, at 0 m and at 15 m depth. Because the particles within each set were released on a regular 1/4 degree grid, if the particles remained immobile over the 1440 time steps of the trajectories, the density would uniformly be 1440. It is not so because the particles were obviously advected by the simulated ocean currents of the model, yet the distribution of the spatial density values peaks at 1440 for both depth levels (distribution were calculated for grid cells between 70°S and 75.25°N). The relative spatial uniformity of these maps is a result of the Lagrangian release scheme and the assembly of forward and backward trajectories. The largest departures from the 1440-value density are found in known energetic oceanic regions with intense horizontal divergence and convergence, such as western boundary currents and along the Equator.

**Fig. 3:**
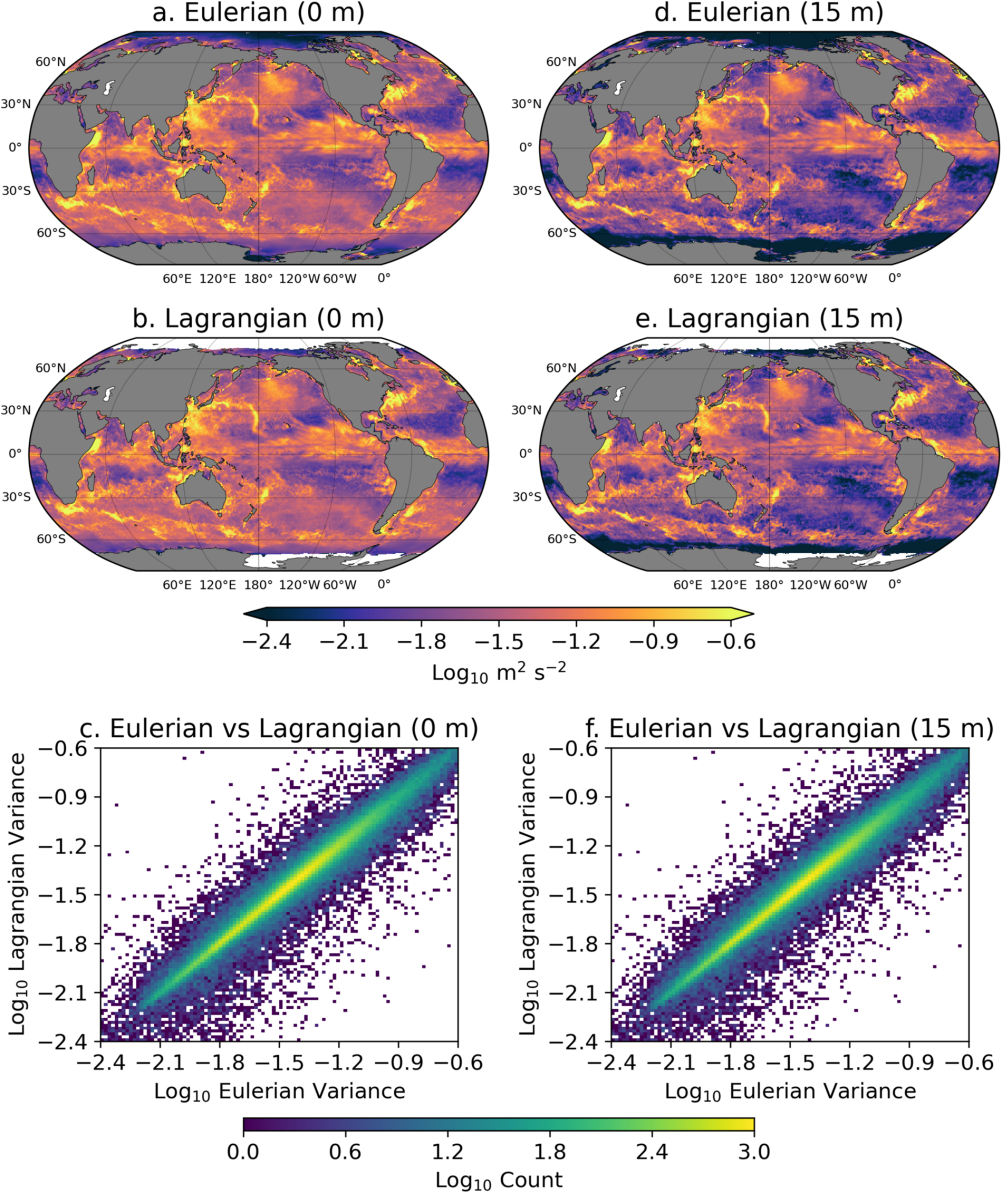
Comparisons of variance of Eulerian and Lagrangian velocities for the time period 2014-01-01 01:00:00 to 2014-03-02 00:00:00 corresponding to the first sets of Lagrangian data. (a,d) Eulerian velocity variance in 1/2 degree longitude-latitude bins at 0 m and at 15 m. (b,d) Lagrangian velocity variance in 1/2 degree longitude-latitude bins at 0 m and at 15 m. (c,f) Two-dimensional histogram of Lagrangian vs Eulerian velocity variance for 0 m and 15 m (in 0.018 ${\log }_{10}$ m^2^ s^−2^ bins).

We now examine the particle velocities calculated from displacements by comparing the velocity variance at both depths, from the Lagrangian particles on one hand, to the velocity variance from the Eulerian velocity fields on the other hand (Fig. 3). The velocity variance is calculated from either all Lagrangian velocities or every other three Eulerian velocities, contained within 1/2 degree bins for the time period 2014-01-01 01:00:00 to 2014-03-02 00:00:00, which corresponds to the first sets of Lagrangian data at 0 m and 15 m. Within that figure, visual examination of panels a and d versus panels d and e shows how the Lagrangian velocities correspond closely to the Eulerian ones. The correlation and regression coefficients between the Lagrangian and Eulerian variance values are 0.95 and 0.78 at 0 m and 0.93 and 0.86 at 15 m (similar statistics are found for the other 60-day periods). The correspondence between Lagrangian and Eulerian velocities is further shown in panels c and f that display two-dimensional histograms of the Eulerian versus Lagrangian binned variance values at 0 m and 15 m, respectively. Velocity variance estimates from Eulerian and Lagrangian perspectives are not expected to strictly correspond, for kinematic reasons that are beyond the scope of this data descriptor paper^18^. However, the close relationships illustrated here constitute an attestation of the ability of the Lagrangian velocity dataset to capture the overall dynamics simulated by HYCOM.

## Usage Notes

We have written a number of Jupyter notebooks in Python to demonstrate some possible usages of this dataset. The notebooks provided leverage the *zarr* nature of the archives stored in an AWS S3 bucket, which when combined with the capabilities of the *xarray* package, allows users to “lazily” open the datasets without the need to download the data to their local computer, until the data are required for some explicit computations.

Seven notebooks are provided in a single GitHub repository, the same repository containing the Python scripts that were used to generate and assemble this dataset, located at https://github.com/selipot/hycom-oceantrack, and archived on Zenodo^19^. The notebooks are succinctly described below.

- tutorials/hycom_eulerian_tutorial.ipynb: In this notebook tutorial, one can learn how to open the Eulerian data from the AWS S3 bucket and how to conduct some simple analysis of the velocity and SSH data. This notebook also provides the necessary code to generate a figure similar to Fig. 1.
- tutorials/hycom_lagrangian_tutorial.ipynb: In this notebook tutorial, one can learn how to access the Lagrangian data and how to conduct some simple analysis of the velocity and SSH data. This notebook also shows how to utilize the grounding mask for particles and subsequently use the *clouddrift* Python package^20^ to convert the original two-dimensional Lagrangian dataset into a ragged array dataset for further Lagrangian analyses.
- tutorials/generate_lagrangian_animation.ipynb: In this notebook, one can learn how to generate a simple animation of particle motions for 60-days.
- tutorials/calculate_velocity_variance.ipynb: In this notebook, one can learn how to generate maps of Eulerian and Lagrangian kinetic energy such as the ones displayed in Fig. 3 of this paper.
- paper-figures/figure1.ipynb: This notebook generates Fig. 1 of this paper.
- paper-figures/figure2.ipynb: This notebook generates Fig. 2 of this paper.
- paper-figures/figure3.ipynb: This notebook generates Fig. 3 of this paper.

## Data Availability

The general numerical code for HYCOM is available from the a consortium which maintains the source code at a GitHub repository at https://github.com/HYCOM. For this HYCOM-OceanTrack dataset^1^, we have created a dedicated and public GitHub repository at https://github.com/selipot/hycom-oceantrackand archived on Zenodo^19^. This repository contains the following: • A suite of Python scripts used to create the cloud-optimized files containing the Eulerian fields of this dataset (See Data Records). • An example Python script showing how the Lagrangian particle advection and field sampling of the model Eulerian fields were conducted using the *Ocean Parcels* software (See Methods). • An example Python script showing how the cloud-optimized files containing the Lagrangian data of this dataset (See Methods) were created. • A suite of Jupyter notebooks written in Python to illustrate possible usages of the dataset (See Usage Notes).
